# Flow cytometric identification of plasmacytoid dendritic cells in acute myeloid leukaemia: Challenges and practical considerations

**DOI:** 10.1111/bjh.70807

**Published:** 2026-09-01

**Authors:** Mariana S. Vieira, Pietro B. Belmonte, Natália A. Marcondes, Raquel Q. Vidal, Flavo B. Fernandes, Liane N. Rotta

**Affiliations:** ^1^ Laboratório Zanol Hospital Moinhos de Vento Porto Alegre Brazil; ^2^ Graduate Program in Health Sciences Federal University of Health Sciences of Porto Alegre (UFCSPA) Porto Alegre Brazil; ^3^ Medicine Program Federal University of Health Sciences of Porto Alegre (UFCSPA) Porto Alegre Brazil; ^4^ Biomedical Sciences Program Federal University of Health Sciences of Porto Alegre (UFCSPA) Porto Alegre Brazil

**Keywords:** flow cytometry, mature plasmacytoid dendritic cell proliferation, plasmacytoid dendritic cell acute myeloid leukaemia

To the Editor,

Mature plasmacytoid dendritic cell proliferation (MPDCP) associated with myeloid neoplasms has been formally recognized in the fifth edition of the World Health Organization (WHO) Classification of Haematolymphoid Tumours.^1^ Within this category, plasmacytoid dendritic cell proliferation associated with acute myeloid leukaemia (pDC‐AML) occurs in approximately 4.9% of AML cases. This entity is clinically relevant because of its association with an adverse prognosis and the growing evidence supporting distinct therapeutic approaches.^1^, ^2^ Given this clinical relevance, accurate identification and quantification of plasmacytoid dendritic cells (pDCs) by flow cytometry are essential, as the diagnosis requires the detection of ≥2% pDCs among leucocytes.^1^

Despite its recent recognition, standard acute myeloid leukaemia (AML) flow cytometry antibody panels and gating strategies often do not adequately address reliable pDC detection. To the best of our knowledge, no specific recommendations are currently available in the literature for the identification and quantification of pDCs in AML. Current literature defines pDCs by bright CD123 and Human Leukocyte Antigen‐DR isotype (HLA‐DR) expression in the absence of lineage‐specific markers such as CD3, CD19, CD56, CD14, CD11c and MPO.^1^, ^2^, ^3^ Expression of pDC‐associated markers, including CD303 and CD304, is also expected, although these are not routinely included in diagnostic panels and may be absent in immature pDCs.^3^, ^4^, ^5^, ^6^ However, in the pathological context of AML, pDCs may exhibit immunophenotypic aberrancies, downregulation of lineage‐associated markers and immature immunophenotypes overlapping with myeloid blasts, which may make pDC identification more challenging.^2^, ^4^, ^5^, ^6^

To characterize the limitations of current diagnostic approaches and explore practical solutions for pDC identification, we retrospectively analysed 504 AML cases from bone marrow (*n* = 369) and peripheral blood (*n* = 135) samples evaluated by flow cytometry between 2020 and 2024 at a specialized haematology laboratory in Porto Alegre, Brazil. Samples were acquired using a DxFLEX flow cytometer (Beckman Coulter, Brea, CA, USA), equipped with three lasers and capable of detecting up to 13 fluorescence parameters and analysed using Kaluza software (Beckman Coulter, version 1.2). Antibody panels and fluorochrome combinations are detailed in Table S1. The gating strategy for pDC identification was based on HLA‐DR and CD123 expression and is illustrated in Figure 1A, while blasts were identified according to the expression of immaturity‐associated markers (CD34, CD117, HLA‐DR and weak CD45) and complemented by lineage‐specific markers when applicable. In a few cases, when the antibody combination initially employed did not allow confident identification or quantification of a specific cell population, additional markers were evaluated according to the diagnostic needs of each case. AML cases were classified according to the WHO category ‘defined by differentiation’ based solely on flow cytometric findings, as molecular data were not available for further classification (Table S2).^1^ This study was approved by the local research ethics committee (UFCSPA, approval no. 6.710.641).

**FIGURE 1:**
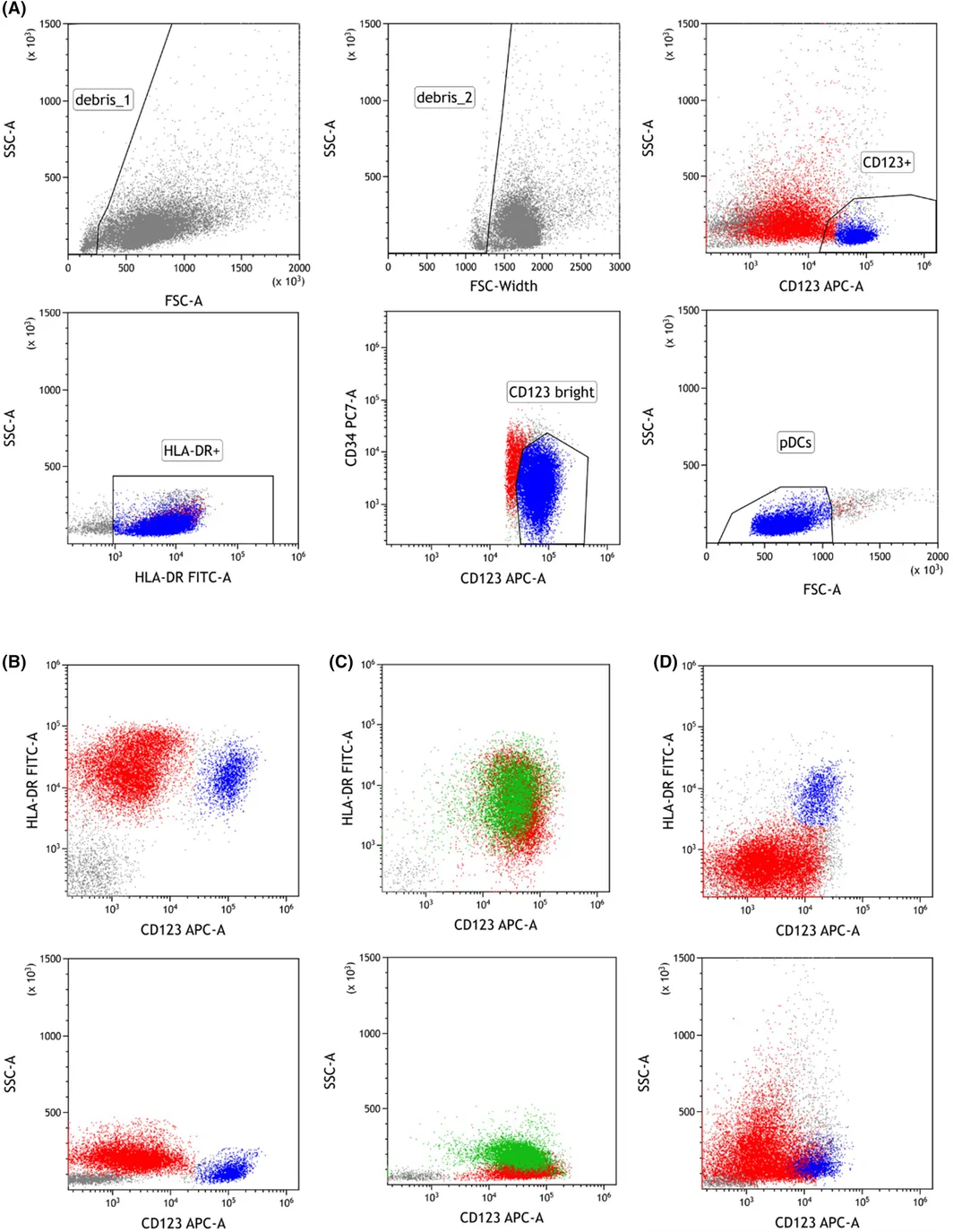
Myeloid blasts are shown in red, monocytic blasts in green and plasmacytoid dendritic cells (pDCs) in blue. Myeloid blasts were identified according to their side scatter (SSC), CD45 expression, CD34 expression and immunophenotypic profile. Monocytic blasts were identified according to their side scatter (SSC), CD45 expression and HLA‐DR expression. (A) Example of the gating strategy applied for pDC identification. After exclusion of debris, pDCs were identified based on expression of HLA‐DR and CD123. Blasts were subsequently distinguished from pDCs using CD34 and CD123 expressions. (B) Expected CD123 expression pattern in blasts and pDCs (bone marrow from patient 1). (C) Myeloid and monocytic blasts exhibiting bright CD123 expression, with intensity similar to that observed in pDCs (peripheral blood from a patient with acute myelomonocytic leukaemia without pDC proliferation). (D) pDCs showing dim CD123 expression, similar to myeloid blasts (bone marrow from patient 15).

Among the cases, 18 (3.57%) were classified as pDC‐AML, while 29 (5.75%) showed pDCs without proliferation (<2% pDCs). The frequency of pDC‐AML observed in our study was comparable to that previously reported in the literature.^2^, ^7^ Additional information on the pDC‐AML cases, including immunophenotype, pDC percentage, sample source (bone marrow or peripheral blood) and AML classification, is provided in Table S2.

Our analysis demonstrated substantial uncertainty in pDC identification when CD123 and HLA‐DR were not simultaneously available, particularly given the immunophenotypic heterogeneity of both blasts and pDCs observed in pDC‐AML. Bright CD123 expression by blasts was observed in 3/18 (16.7%) pDC‐AML cases, 4/29 (13.8%) AML without pDC proliferation cases and 63/457 (13.8%) AML without pDCs cases (Figure 1C). Among AML without pDC proliferation and AML without pDCs cases with bright CD123 expression by blasts, monocytic differentiation was present in 2/4 (50.0%) and 23/63 (36.5%) cases respectively. This feature was not present among pDC‐AML cases with bright CD123 expression by blasts. Conversely, weak CD123 expression by pDCs was observed in 5/18 (27.8%) pDC‐AML cases (Figure 1D) and 3/29 (10.3%) AML without pDC proliferation cases. These findings highlight the potential overlap in CD123 expression between these two populations. In such scenarios, markers preferentially expressed by blasts, or expressed at higher levels in this population, such as CD33, CD13 and CD117, may be helpful in distinguishing blasts from pDCs in some cases.

In addition, the choice of fluorochrome for CD123 significantly influenced pDC detection, as lower fluorescence intensity impaired discrimination between pDCs and myeloid blasts. This limitation may be particularly relevant in cases with weak CD123 expression by pDCs or bright CD123 expression by blasts, where accurate discrimination between these populations can be challenging. In our study, CD123 conjugated to allophycocyanin (APC) was extensively used and provided adequate separation between pDCs and myeloid blasts in most cases, supporting its application for pDC identification in this setting.

We observed heterogeneous pDC immunophenotypes reflecting different stages of maturation. Because immunophenotypic features vary according to pDC maturation stage, distinguishing blasts from immature pDCs requires particular caution, as these populations may share expression of CD13, CD33, CD34 and CD117.^5^ To provide additional context for the interpretation of the pDC immunophenotype in pDC‐AML cases, we therefore report the proportion of immature pDCs (Stages I and II), identified by CD34 expression and/or absence of CD4, in Table S2.

We also identified abnormal pDC immunophenotypes, including dim or partial CD56 expression. Assessment of CD56 expression remains important for excluding blastic plasmacytoid dendritic cell neoplasm (BPDCN), in which CD56 is typically brightly expressed, whereas dim expression may be observed in pDC‐AML.^3^, ^5^, ^6^ These findings highlight the need for cautious gating strategies to avoid inadvertent exclusion of pDC subsets, which may lead to underestimation of their quantification and diagnostic misclassification.

In addition, distinguishing pDCs from monocytic precursors may be challenging in some cases, as these populations may share overlapping features such as expression of CD36, HLA‐DR and CD123; dim expression of CD4 and CD33; and similar CD45 profile (Figure 2). In this setting, bright CD123 expression and the absence or dim expression of CD64 in pDCs may aid in the differential diagnosis. The use of pDC‐specific markers, such as CD303, CD304 and TCL1, may also be helpful, although these markers may be absent in immature or aberrant pDCs.^5^, ^6^

**FIGURE 2:**
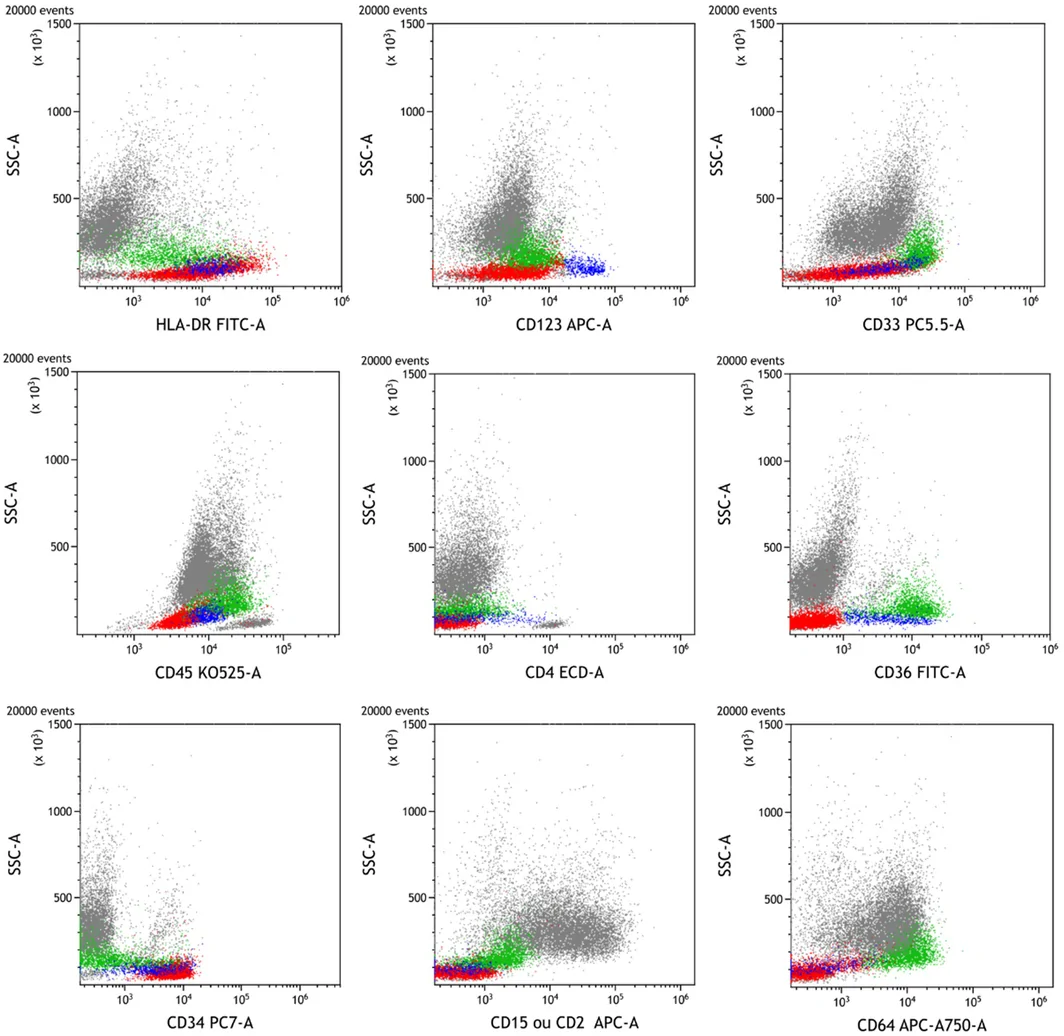
Comparative immunophenotypic expression of blasts (red), monocytes (green) and plasmacytoid dendritic cells (blue) across different markers (bone marrow, patient 16).

Evaluation of CD11c expression may further assist in distinguishing pDCs from transitional dendritic cells (tDCs), as both populations express HLA‐DR, CD123 and CD303, whereas only tDCs express CD11c.^8^ However, the expression profile of CD11c in pDCs in the pathological context of pDC‐AML remains poorly characterized and warrants further investigation.

Based on our findings, we recommend the routine inclusion of CD123 and HLA‐DR within the same tube in AML diagnostic workflows, with careful fluorochrome selection to optimize pDC detection. This approach is consistent with national standardization initiatives advocating the combined use of HLA‐DR and CD123 in AML diagnosis.^9^ Notably, some widely used diagnostic panels for AML neither mandate nor include the simultaneous assessment of CD123 and HLA‐DR within the same tube, which may compromise pDC identification.^10^, ^11^

The selection of additional markers within the same tube is equally important to ensure accurate pDC identification and quantification, as CD123 and HLA‐DR alone may be insufficient to distinguish pDCs from other cell populations. Based on our data, we propose the inclusion of CD34, CD117, CD33 and CD64 as a minimal set of additional markers to improve specificity and reduce misclassification with myeloid and progenitor compartments. In addition, CD56 and CD11c are important for the discrimination of pDCs from BPDCN and tDCs respectively.

CD4 may be included as a supportive marker for assessing pDC maturation status, although it does not provide additional clinical value in routine diagnostic settings. Although not essential for diagnostic purposes, CD303 and CD304 may be useful when available to support lineage confirmation. Nevertheless, these markers should be interpreted with caution, as their absence does not exclude pDCs, particularly in immature or immunophenotypically aberrant populations.

When panel modification is not feasible, the addition of a dedicated pDC‐focused tube represents a practical alternative. To support the implementation of these strategies, further studies are needed to develop standardized flow cytometry protocols for pDC assessment in AML and enable accurate diagnosis of pDC‐AML.

## AUTHOR CONTRIBUTIONS

**Mariana S. Vieira:** Investigation; methodology; data curation; formal analysis; writing – original draft; writing – review and editing. **Natália A. Marcondes:** Conceptualization; writing – review and editing; supervision. **Pietro B. Belmonte:** Writing – original draft; writing – review and editing. **Raquel Q. Vidal:** Investigation; writing – review and editing. **Flavo B. Fernandes:** Writing – review and editing. **Liane N. Rotta:** Conceptualization; project administration; writing – review and editing; writing – original draft.

## FUNDING INFORMATION

This work was supported by Coordination for the Improvement of Higher Education Personnel (CAPES)—Finance code 001.

## CONFLICT OF INTEREST STATEMENT

The authors have no conflicts of interest to disclose.

## Supporting information


**Table S1.** Antibody panels used for flow cytometric evaluation of AML cases.

**Table S2.** Clinical and immunophenotypic features of pDC‐AML cases.

## Data Availability

Data are available upon request.
