# Supplementary material for: Flow cytometric identification of plasmacytoid dendritic cells in acute myeloid leukaemia: Challenges and practical considerations
Source: Br J Haematol. 2026 Sep 1;209(5):2346–50. doi: 10.1111/bjh.70807 (PMC13748365; doi:10.1111/bjh.70807)
Supplement: Supplementary file 1 [file BJH-209-2346-s001.docx]

**Table S1. Antibody panels used for flow cytometric evaluation of AML cases**

|  | **Markers** | | | | | | | | | |
| --- | --- | --- | --- | --- | --- | --- | --- | --- | --- | --- |
|  | **FITC** | **PE** | **ECD** | **PC5.5** | **PC7** | **APC** | **APC-A700** | **APC-A750** | **PB** | **KO** |
| **Tube 1** | nTdT* | cyMPO | - | - | - | cyCD79a | cyCD22* | cyCD3 | - | cyCD45 |
| **Tube 2** | CD65 | CD14 | CD13 | CD33 | CD34 | CD117 | CD7 | CD11b | CD16 | CD45 |
| **Tube 3** | HLA-DR | CD10 | CD4 | - | CD34 | CD123 | CD56 | CD19 | CD38 | CD45 |
| **Tube 4** | CD36 | CD71 | CD24 | CD61 | CD34 | CD15 | - | CD64 | CD2 | CD45 |

Abbreviations: * antibodies evaluated only in selected cases; cy, cytoplasmic expression.
