# Supplementary material for: Flow cytometric identification of plasmacytoid dendritic cells in acute myeloid leukaemia: Challenges and practical considerations
Source: Br J Haematol. 2026 Sep 1;209(5):2346–50. doi: 10.1111/bjh.70807 (PMC13748365; doi:10.1111/bjh.70807)
Supplement: Supplementary file 2 [file BJH-209-2346-s002.docx]

**Table S2. Clinical and immunophenotypic features of pDC-AML cases**

| **Patient** | **Material** | **WHO classification**^a^ | **% pDC** | **CD123 expression** | | **% pDCs CD4- and/or CD34+** | **Immunophenotypic profile of blasts** | | | | |
| --- | --- | --- | --- | --- | --- | --- | --- | --- | --- | --- | --- |
|  |  |  |  | **Weak on pDCs** | **Bright on blasts** |  | **CD34** | **CD117** | **HLA-DR** | **CD13** | **CD33** |
| **1** | BM | [without maturation](https://tumourclassification.iarc.who.int/chaptercontent/63/52) | 8.4 | no | no | 30.5 | positive | positive | positive | partial | bright |
| **2** | BM | [without maturation](https://tumourclassification.iarc.who.int/chaptercontent/63/52) | 7.6 | no | no | 52.7 | positive | positive | partial | partial | partial |
| **3** | BM | [myelomonocytic](https://tumourclassification.iarc.who.int/chaptercontent/63/55) | 10.5 | no | no | 48.8 | positive^b^ | positive^b^ | positive^b^ | partial^b^ | bright^b^ |
|  |  |  |  |  |  |  | partial^c^ | partial^c^ | positive^c^ | partial^c^ | bright^c^ |
| **4** | PB | [without maturation](https://tumourclassification.iarc.who.int/chaptercontent/63/52) | 5.8 | no | no | 94.0 | positive | partial | positive | partial | partial |
| **5** | BM | [minimal differentiation](https://tumourclassification.iarc.who.int/chaptercontent/63/51) | 3.2 | no | no | 19.0 | partial | partial | positive | positive | negative |
| **6** | BM | [without maturation](https://tumourclassification.iarc.who.int/chaptercontent/63/52) | 41.1 | yes | no | 37.9 | positive | partial | positive | positive | partial |
| **7** | BM | [without maturation](https://tumourclassification.iarc.who.int/chaptercontent/63/52) | 3.8 | no | yes | 79.3 | positive | positive | bright | partial | positive |
| **8** | BM | [minimal differentiation](https://tumourclassification.iarc.who.int/chaptercontent/63/51) | 14.4 | no | yes | 68.5 | positive | partial | partial | partial | partial |
| **9** | BM | [without maturation](https://tumourclassification.iarc.who.int/chaptercontent/63/52) | 10.2 | no | no | 41.0 | positive | partial | positive | partial | partial |
| **10** | BM | [without maturation](https://tumourclassification.iarc.who.int/chaptercontent/63/52) | 8.5 | no | no | 49.7 | positive | partial | partial | partial | positive |
| **11** | BM | [without maturation](https://tumourclassification.iarc.who.int/chaptercontent/63/52) | 14.3 | no | yes | 54.5 | positive | partial | partial | partial | negative |
| **12** | BM | [without maturation](https://tumourclassification.iarc.who.int/chaptercontent/63/52) | 2.6 | no | no | 29.7 | positive | positive | positive | partial | positive |
| **13** | BM | [minimal differentiation](https://tumourclassification.iarc.who.int/chaptercontent/63/51) | 15.8 | no | no | 32.2 | positive | partial | partial | partial | partial |
| **14** | BM | [minimal differentiation](https://tumourclassification.iarc.who.int/chaptercontent/63/51) | 6.1 | no | yes | 40.6 | positive | partial | positive | partial | negative |
| **15** | BM | [without maturation](https://tumourclassification.iarc.who.int/chaptercontent/63/52) | 6.9 | yes | no | 97.8 | negative | partial | negative | partial | bright |
| **16** | BM | [without maturation](https://tumourclassification.iarc.who.int/chaptercontent/63/52) | 8.8 | yes | no | 41.2 | positive | positive | positive | partial | partial |
| **17** | BM | [without maturation](https://tumourclassification.iarc.who.int/chaptercontent/63/52) | 5.7 | yes | no | 53.4 | partial | partial | positive | partial | positive |
| **18** | BM | [without maturation](https://tumourclassification.iarc.who.int/chaptercontent/63/52) | 2.8 | yes | no | 44.7 | positive | positive | positive | partial | positive |

Abbreviations: BM, blood marrow; PB, peripheral blood; ^a^, acute myeloid leukaemia defined by differentiation according to the 5th edition of the WHO Classification; [without maturation](https://tumourclassification.iarc.who.int/chaptercontent/63/52), [acute myeloid leukaemia without maturation](https://tumourclassification.iarc.who.int/chaptercontent/63/52); myelomonocytic, [acute myelomonocytic leukaemia](https://tumourclassification.iarc.who.int/chaptercontent/63/55); [minimal differentiation](https://tumourclassification.iarc.who.int/chaptercontent/63/51), [acute myeloid leukaemia with minimal differentiation](https://tumourclassification.iarc.who.int/chaptercontent/63/51); ^b^, myeloid blasts; ^c^, monocytic blasts;
